# The association of food quality score and cardiovascular diseases risk factors among women: A cross-sectional study

**DOI:** 10.15171/jcvtr.2019.39

**Published:** 2019-08-29

**Authors:** Manije Darooghegi Mofrad, Nazli Namazi, Bagher Larijani, Nick Bellissimo, Leila Azadbakht

**Affiliations:** ^1^Department of Community Nutrition, School of Nutritional Sciences and Dietetics, Tehran University of Medical Sciences, Tehran, Iran; ^2^Students’ Scientific Research Center (SSRC), Tehran University of Medical Sciences (TUMS), Tehran, Iran; ^3^Diabetes Research Center, Endocrinology and Metabolism Clinical Sciences Institute, Tehran University of Medical Sciences, Tehran, Iran; ^4^Endocrinology and Metabolism Research Center, Endocrinology and Metabolism Clinical Sciences Institute, Tehran University of Medical Sciences, Tehran, Iran; ^5^School of Nutrition, Ryerson University, Toronto, Canada; ^6^Department of Community Nutrition, School of Nutrition and Food Science, Isfahan University of Medical Science, Isfahan, Iran

**Keywords:** Food Quality Score, Cardiovascular Risk Factors, Obesity, Diabetes, Dyslipidemia

## Abstract

***Introduction:*** Limited studies are available regarding the relationship between Food Quality Score (FQS) and cardiovascular disease (CVD) risk factors. Thus, this study was aimed to investigate the association of FQS with CVD risk factors in women.

***Methods:*** This cross-sectional study was carried out among 368 women aged 20-50 years who randomly selected from health centers across Tehran, Iran. Dietary intake was collected using a reliable and validated food frequency questionnaire (FFQ). The FQS includes vegetables, fruits, whole grains, yogurt, nuts and legumes, coffee, refined grains, desserts and ice cream, sugar-sweetened beverages, red meats, fried food consumed outside the home, processed meats, potato and potato chips. Standard methods were used to assess blood pressure, biochemical and anthropometric measures. Multivariate logistic regression was used to examine the association between FQS and CVD risk factors.

***Results:*** Participant mean age and body mass index (BMI) were 30.7 ± 6.9 years and 24.3 ± 4.0 kg/m2, respectively. After taking potential confounders into account, FQS had no significant association with risk of overweight and obesity [Odds ratio (OR): 1.1, 95% confidence interval (CI): 0.68, 1.8; *P * = 0.683], diabetes (OR: 0.62, 95% CI: 0.22, 1.74; *P * = 0.374), metabolic syndrome (OR: 0.36, 95% CI: 0.10, 1.32; *P * = 0.127), hypercholesterolemia (OR: 0.54, 95% CI: 0.29, 1.01; *P*= 0.051), or hypertriglyceridemia (OR: 1.63, 95% CI: 0.71, 3.70; *P * = 0.244).

***Conclusion:*** The results showed that FQS was not significantly associated with CVD risk factors among women. Prospective cohort studies are warranted to confirm our findings.

## Introduction

Cardiovascular diseases (CVDs) are the main cause of worldwide death, which are rising in both developing and developed countries.^1^ In developed countries, approximately 43% of men and 55% of women have CVDs.^2^ In the past decade, CVDs have been recognized as the main cause of mortality in Iran.^3^ Dyslipidemia, obesity and metabolic syndrome increase the risk of CVDs.^4^ Despite developed countries,^5^ CVDs risk factors have been indicated to be more prevalent among women compared with men in Iran.^6^

Physical activity and healthy eating patterns can decrease the risk of CVDs. Recently, the examination of the whole diet pattern has been applied to study the role of diet on disease prevention.^7^ Several dietary scores such as Dietary Approaches to Stop Hypertension (DASH) score and Dietary Diversity Score (DDS) have been developed to measure adherence to dietary patterns or recommendations.^8^ Many of these scores may be associated with the reduced prevalence of CVD and mortality.^9-11^ However, most of these diet-based dietary quality indices consider a combination of both nutrients and foods. The evaluation of nutrient intake requires entering food intake data into nutrient database, which are prone to bias. Food-based scores do not require software or access to specific databases to perform the nutrient analysis.^12^ Several studies have demonstrated that food-based diet quality scores linked with CVD risk factors, such as oxidative stress, obesity, low-density lipoprotein cholesterol (LDL-C) levels, and blood pressure.^13,14^ Only two studies have assessed the relationship between Food Quality Score (FQS) and risk of metabolic syndrome^15^ and coronary heart disease (CHD).^12^ These studies showed FQS decreased the risk of CHD,^12^ while there was no significant association with the risk of metabolic syndrome.^15^ Moreover, one study previously identified foods which were components of FQS that associated with long-term weight loss.^16^

Based on our knowledge, no study exists to examine the association between FQS and cardiovascular risk factors in Iran. Additionally, it was shown that risk factors of CVDs are more prevalent in Iranian women compared with men.^6^ Therefore, we examined the association of FQS with cardiometabolic risk factors among women in Tehran, Iran.

## Materials and Methods

### Participants and sampling

This cross-sectional study included 368 women between 20 and 50 years who were recruited from health centers across Tehran, Iran, in 2018. Participants inclusion was conducted by a random sampling method. The sample size was estimated using different cardiometabolic variables such as lipid factors, metabolic syndrome factors and serum fasting blood sugar (FBS). However, because the sample size calculated by FBS was higher compared with the other variables, it was considered as the dependent variable.^17^ The sample size was determined using a standard deviation (SD) =24.4, d =2.5, α = 0.05 and β = 0.8 with the following formula: N = [(Z _1-α_/2)^2^× S^2^]/d^2^.

To be included, participants were between 18 and 50 years old, not lactating or pregnant, with no history of malignant diseases, and not following a specialized diet. In the current study, participants who had energy intakes outside the range of 500–3500 kcal/d were excluded. All participants should complete the informed consent form before the study.

### Dietary assessments

Dietary intake was evaluated using a reliable and valid 168-item semi-quantitative FFQ.^18^ All FFQs were completed by an expert dietitian through face-to-face interviews. The portion sizes were converted to grams using homebred scales. Mean nutrient and energy intakes were estimated using a modified version of Nutritionist IV software for Iranian foods (version 7.0; N-Squared Computing, Salem, OR, USA).

### FQS calculation

We used the method defined by Fung et al,^12^ for scoring food quality. The components of FQS includes vegetables, fruits, whole grains, yogurt, nuts and legumes, coffee, refined grains, desserts and ice cream, sugar-sweetened beverages, red meats, fried food consumed outside the home, processed meats, potato and potato chips. Yogurt, nuts and legumes, fruits, whole grains, vegetables, and coffee were classified as healthy foods or food groups; red meats, refined grains, processed meats, sugar-sweetened beverages, fried foods prepared away from home, potatoes, potato chips, and desserts and ice cream were classified as unhealthy foods or food groups. We then ranked participant intakes into tertiles and assigned scores between 1 and 3 for healthy foods or food groups, and assigned reversed tertile rankings (scores between 3 and 1) for unhealthy foods or food groups. The scores from each food group were assembled and overall score ranged between 14 to 42. A higher score expresses a healthier dietary pattern.

### Biochemical assessment

Biochemical parameters were measured following a 12-hour overnight fast. FBS was measured on the blood sampling day and the remaining serum was stored at -80°C until analyzed. Serum levels of FBS, total cholesterol (TC), high-density lipoprotein cholesterol (HDL-C), triglyceride (TG), and low-density lipoprotein cholesterol (LDL-C) were quantified using commercially available enzymatic reagents (Pars Azmoon, Tehran, Iran) adapted to an auto-analyzer system (Selectra E, Vitalab, Holliston, the Netherlands).

### Assessment of Anthropometric Indices

Using digital scale [SECA 813; Seca, Hamburg, Germany], weight measurement was carried out to the nearest 100 g, while participants were barefoot and wore minimal clothing. Height was measured using a non-stretchable tape with participants standing in an upright position, barefoot, and shoulders in a neutral position. Waist circumference (WC) was measured at the narrowest part of the waist, and at the body mass index (BMI) was determined by dividing weight in kilograms by height in meters squared.

### Metabolic syndrome definition

According to the National Cholesterol Education Program Adult Treatment Panel III (ATP III) guidelines,^19^ people with three or more of the following criteria have Metabolic Syndrome (MS): (1) high levels of FBS (≥100 mg/dL), (2) WC >88 cm in women, (3) increased levels of TG (≥150 mg/dL), (4) low levels of HDL-C (<50 mg/dL), and (5) high blood pressure (systolic ≥130 mm Hg and diastolic ≥85 mm Hg).

### Assessment of other variables

Systolic and diastolic blood pressure (SBP & DBP) were measured on the left arm in duplicate, after a minimum of 5 minutes of rest in a quiet sitting position. Trained technicians measured SBP and DBP, which were determined as the appearance of clear tapping sounds (first Korotkoff phase) and vanishing of sound (fifth Korotkoff phase), respectively. The average of the two blood pressure measurements was calculated and used in the analysis. Several questions regarding education, occupation, income, family number, home ownership, car ownership, travel outside and within the country, having modern furniture at home, and the number of rooms at home were used to determine socioeconomic status. Demographic characteristics were also recorded using a demographic questionnaire. Physical activity was reported as metabolic equivalents (METs) in hours per day. To estimate the METs for each patient, each physical activity duration (h/d) was multiplied by its METs coefficient using standard tables.^20^

### Statistical analysis

Demographic characteristics, dietary intake, blood pressure and biochemical biomarkers were compared based on the median-split of FQS (group 1 (G1) <28, group 2 (G2) ≥ 28). Student’s *t*-test and chi-square test were applied for comparing means ± SDs of continuous variables and numbers and percentages of categorical variables between FQS groups, respectively. Both crude and adjusted models (age, energy intake, physical activity, socio-economic status and BMI) were reported to compare quantitative variables across the FQS medians. To compare the pre-determined parameters between groups, analysis of covariance (one-way ANCOVA) was used.

Multivariate logistic regression as crude and controlled models were used to examine the association of FQS with cardiometabolic risk factors. Age, energy intake, physical activity, socioeconomic status, and BMI were adjusted. Group 1 was considered the reference group. The cut off points for high risk of CVD were as follows according to ATP III criteria^21^: high TG (>150 mg/dL), FBS (>100 mg/dL), TC(> 200 mg/dL ), LDL (>130 mg/dL), SBP (>140 mm Hg), DBP (>90 mm Hg). All statistical analyses were conducted by SPSS software (version 18, SPSS Inc., Chicago, IL, USA). *P* < 0.05 was defined statistically significant.

## Results

A total of 368 women with an average age of 30.7 ± 6.9 years were included in the analysis. The general participant characteristics by median-split FQS are presented in Table 1. Compared with group 1, group 2 were older, more likely to be married, and higher education (*P* < 0.05). Other general characteristics of study participants were not significantly different across groups of FQS.

**Table 1:** General characteristics of study participants between FQS groups

**Variable**	**Total patients**	**FQS medians**		***P*** ** value†**
		**G1 ≤ 28 (n=154)**	**G2 >28 (n=214)**	
Age (y)	30.7 ± 6.9	29.6 ± 6.0*	31.5 ± 7.5	0.002
Socioeconomic status	31.99 ± 6.18	33.26 ± 5.95	31.08 ± 6.20	0.227
Marital status (%)				0.022
Married	229 (62)	92 (40)	137 (60)	
Single	131 (36)	62 (47)	69 (53)	
Divorced	8 (2)	0 (0)	8 (100)	
Education status (%)				0.003
Illiterate	33 (9)	12 (36)	19 (64)	
Diploma	169 (46)	53 (31)	116 (69)	
Universal	166 (45)	99 (57)	77 (43)	
BMI (kg/m^2^)	24.3 ± 4.0	23.4 ± 3.8	24.9 ± 4.1	0.067
Physical activity (Met.h/d)		26.8 ± 3.85	27.6 ± 4.3	0.285
Multi vitamin use				0.129
Yes	52 (14)	26 (50)	26 (50)	0.129
No	316 (86)	128 (40)	188 (60)	0.129

FQS: food quality score; BMI: body mass index. *Data were reported as mean±SD. †Obtained from *t* test for continuous variables and χ2 test for categorical variables.

Comparison of dietary intakes between the median-split FQS is provided in Table 2. After adjustment for energy intake, participants in group 2 consumed higher amounts of dietary carbohydrate, vitamin A, vitamin C, calcium, potassium, and magnesium (*P* < 0.05). There were no significant differences between groups for dietary fat, mono unsaturated fatty acids (MUFA), saturated fatty acids (SFA), poly unsaturated fatty acids (PUFA), vitamin B6 and zinc (*P* > 0.05). Moreover, participants in group 2 consumed lower protein, cholesterol, folic acid, thiamine and vitamin B12 (*P* < 0.05).

**Table 2:** Energy-adjusted of dietary intakes between FQS groups

**Variable**	**FQS medians**		***P*** ** value†**
	**G1 ≤ 28 (n=154)**	**G2 >28 (n=214)**	
Protein (g/day)	94.7±16.3*	87.6±16.1	<0.001
Carbohydrate (g/day)	369.61±39.83	382.60±39.49	0.003
Fat (g/day)	78.26±15.01	77.35±14.92	0.575
Cholesterol (mg/day)	268.64±124.22	217.68±123.46	<0.001
SFA (g/day)	24.95±6.53	24.64±6.49	0.946
MUFA (g/day)	25.13±5.37	24.25±5.33	0.128
PUFA (g/day)	19.02±8.47	19.54±7.13	0.501
Folic acid (μg/day)	331.41±115.4	329.92±114.83	0.049
Vitamin A (RAE/day)	1279.46±887.08	1494.7±746.06	0.008
Thiamine (mg/day)	2.37±0.4	2.25±0.4	0.009
Vitamin B6 (mg/day)	1.99±0.61	1.98±0.51	0.749
Vitamin B12 (μg/day)	4.45±1.65	3.86±1.63	0.001
Vitamin C (mg/day)	123±74.7	159.67±74.31	<0.001
Calcium (mg/day)	997.83±292.12	1078.4±290.38	0.011
Magnesium (mg/day)	277.76±57.33	303.68±57.05	<0.001
Potassium (mg/day)	3272.95±862.84	3664.39±857.82	<0.001
Zinc (mg/day)	10.43±2.77	9.90±2.76	0.077
Fruit (g/day)	180.46±220.39	357.32±219.13	<0.001
Vegetable (g/day)	329.67±219.27	381.08±217.96	0.03
Nut and legumes (g/day)	12.63±14.89	18.54±14.77	<0.001
Whole grains (g/day)	38.41±58.69	57.88±58.36	0.002
Yogurt (g/day)	115.73±114.91	145.79±114.25	0.015
Sugar sweetened beverage (g/day)	77.59±79.04	57.58±78.55	0.019
Red meat (g/day)	61.22±43.43	41.31±43.15	<0.001
Processed meat (g/day)	7.99±10.52	4.55±10.47	0.003
Refined grains (g/day)	483.01±175.72	408.34±174.66	<0.001
Desserts and ice cream (g/day)	34.89±27.25	28.62±27.06	0.032
Potato (g/day)	28.89±23.95	22.46±23.84	0.013
Potato chips (g/day)	15.17±12.53	12.72±12.46	0.069
Coffee (g/day)	72.60±53.36	111.02±53.10	<0.001
Fried food from outside the home(g/day)	15.84±12.16	8.98±12.08	<0.001

FQS: food quality score; SFA: saturated fatty acid; PUFA: poly unsaturated fatty acid; MUFA; mono unsaturated fatty acid; RAE: Retinol activity equivalents. * Data were reported as mean ± SD. † ANCOVA (adjusted for energy intake).

As presented in Table 2, intake of fruits, nuts and legumes, coffee, vegetable, yogurt, and whole grains was significantly greater in the highest FQS group (*P* < 0.05). In addition, participants in the higher FQS group consumed less red meat, refined grains, sugar-sweetened beverages, processed meat, desserts and ice cream, and potatoes (*P* < 0.05). However, no significant differences were observed between the highest versus lowest FQS groups for potato chips intake (*P* > 0.05).

Crude and adjusted models for anthropometric indices, biochemical markers, and blood pressure between FQS groups are summarized in Table 3. There were not significant differences in SBP, DBP, FBS, HDL-C, TC, and TG between groups after adjustment for known confounders (*P* < 0.05). Women in group 2 had lower LDL-C compared with group 1 (*P* <0.05).

**Table 3:** Mean and standard deviation of biochemical markers and blood pressure between FQS groups

**Variables**	**FQS medians**		***P*** ** value**
	**G1 ≤ 28 (n=154)**	**G2** **>28 (n=214)**	
SBP (mm Hg)			
Crude model	11.44±0.87*	11.42±1.06	0.009
Model 1	11.39±1.01†	11.46±1.00	0.521
DBP (mm Hg)			
Crude model	7.86±0.61	7.77±0.64	0.495
Model 1	7.84±0.64	7.78±0.64	0.426
FBS (mg/d)			
Crude model	88.39±11.03	91.18±31.45	0.021
Model 1	87.36±23.2	91.91±22.96	0.073
LDL-C (mg/dL)			
Crude model	84.13 ± 17.40	77.62 ± 20.14	<0.001
Model 1	82.78 ± 19.00	78.32 ± 18.7	0.037
HDL-C (mg/dL)			
Crude model	48.75 ± 7.75	47.46 ± 10.13	0.005
Model 1	48.44 ± 9.42	47.62 ± 9.35	0.446
Cholesterol (mg/dL)			
Crude model	178.18±36.35	171.19±32.89	0.24
Model 1	177.49±34.99	171.69±34.81	0.125
TG (mg/dL)			
Crude model	95.37±47.41	104.54±64.28	0.001
Model 1	103.59±55.34	104.14±54.85	0.171

FQS: food quality score; FBS: fasting blood sugar; LDL-C: low density lipoprotein-cholesterol; HDL-C: high density lipoprotein-cholesterol TG: triglyceride; SBP: systolic blood pressure; DBP: diastolic blood pressure * Obtained from one-way ANOVA † Obtained from ANCOVA (adjusted for age, energy intake, socioeconomic status).

Multiple-adjusted odds ratio (OR) and 95% confidence intervals (CI) of CVD risk factors between FQS groups are provided in Table 4. There were no differences between groups in FBS (*P* = 0.372), TG (*P* = 0.244), and TC (*P* = 0.051). Furthermore, higher FQS were not associated with obesity and overweight (*P* = 0.683) or metabolic syndrome (*P* = 0.127).

**Table 4:** Multiple-adjusted odds ratio (OR) and 95% confidence intervals (CI) between FQS groups

**Variable**	**FQS groups**		***P*** ** value**
	**G1 ≤ 28** **(n=154)**	**G2 >28** **(n=214)**	
FBS (>100 mg/dL)			
Crude*	1.00	1.22 (0.57, 2.57) †	0.602
Model 1**	1.00	0.62 (0.22, 1.74)	0.372
TG (>150 mg/dL)			
Crude	1.00	2.23 (1.11, 4.47)	0.023
Model 1	1.00	1.63 (0.71, 3.70)	0.244
Cholesterol (>200 mg/dL)‏			
Crude	1.00	0.73 (0.42, 1.26)	0.27
Model 1	1.00	0.54 (0.29, 1.00)	0.05
MS, %			
Crude	1.00	0.56 (0.19, 1.63)	293
Model 1	1.00	0.36 (0.10, 1.32)	0.127
Obesity and overweight			
Crude	1.00	1.2 (0.76, 1.88)	0.421
Model 1	1.00	1.1 (0.68, 1.8)	0.683

FQS: food quality score; FBS: fasting blood sugar; MS: Metabolic syndrome; TG: triglyceride; LDL-C: low density lipoprotein-cholesterol † Odd ratio (95% Confidence Interval). * Not adjusted for any variables. **Adjusted for age, BMI, socioeconomic status, energy intake.

## Discussion

In the present study, we could not find an association between FQS and risk factors of CVDs. In line with our finding, a recent cross-sectional study failed to find a significant relationship between FQS and risk of metabolic syndrome.^15^ In addition, another study using the Elderly Dietary Index, which comprises of 10 food components, did not find any significant associations with prevalence of diabetes, hypertension, and hypercholesterolemia.^22^ In contrast to our finding, one study showed that 1 SD increase in FQS was associated with lower risk of CVDs compared with the Dietary Approach to Stop Hypertension (DASH), alternative Mediterranean diet score (aMed), and Alternative Healthy Eating Index-2010 (AHEI-2010).^12^ In addition, Mozaffarian et al identified food components of FQS were associated with lower long-term weight gain.^16^ However, the foregoing studies were cohorts with large follow-up duration and showed long-term weight gain changes.

Differences among studies may been explained by the variation in carbohydrate intake among study participants. In our study, participants in the highest FQS group consumed significantly higher dietary carbohydrate. Moreover, whole grain consumption was low, while the intake of refined grain was higher in this study population. Whole grains are rich in fiber and nutrients that contribute to improvements in cardiovascular and metabolic function.^23^ Refined grains are rich sources of energy and contain small amounts of nutrients. According to some reports, refined grain intake has been related to risk of metabolic syndrome^24^ and stroke.^25^ Refined grains have high glycemic load, and several studies revealed that glycemic load is positively related to poorer glycemic control or metabolic risk.^26^

One recent meta-analysis found that high vegetable and fruit intakes reduce CVD risk.^27^ Another meta-analysis showed that higher nut consumption was associated with a reduced risk of total CVD, total CHD, CVD mortality, CHD mortality, all-cause mortality, and sudden cardiac death.^28^ In addition, a meta-analysis showed that legume consumption was associated with declined risk of CVD.^29^ Furthermore, Khosravi-Boroujeni et al reported that potato consumption was positively associated with high fasting blood glucose levels and diabetes mellitus, and low HDL-C.^30^ However, another review failed to show a significant association between potato consumption and cardiovascular mortality.^31^ In an epidemiologic study, high processed and red meat intakes were associated with increased BMI and SBP.^32^ However, results from a meta-analysis of randomized clinical trials showed that consumption of red meat did not affect blood pressures, blood lipids and lipoproteins.^33^ This inconsistency may be due to differences in fat content among the different studies. Further, we did not find any significant association between FQS and CVD risk factors in our study. This may have been due to the low number of participants in this study with elevated CVD risk factors.

The possible mechanisms for the association between FQS and cardiovascular risk factors are largely unknown. This association has originated from the additive effects of all components of FQS rather than on an individual nutrient or food group. Antioxidant compounds and polyphenols which are high in fruits, vegetables, nuts, legumes, whole grains and coffee might enhance the antioxidant capacity of serum, increase the formation of endothelial prostacyclin, support against in vivo lipid peroxidation, and suppress platelet aggregation.^34^ Folate, which is abundant in green leafy vegetables, may decrease plasma homocysteine concentrations, a suggested marker for arterial endothelial dysfunction.^35^ Furthermore, according to prior investigations, soluble fibers have shown to play cardioprotective roles by lowering serum levels of cholesterol.^36^ Several minerals, such as magnesium have been shown to improve endothelial function.^37^ The cardioprotective mechanisms of nuts include cholesterol lowering, reduced inflammation, and improved vascular health.^38^ Consumption of sugar sweetened beverages and starchy foods lead to rapid glycemia. Many studies have identified an association between high glycemic load and glycemic index diets and CVD risk factors such as low HDL-C and high TGs.^39^ However, in this study scores for refined grains, potatoes, potato chips, red meat, fried foods, sugar-sweetened beverages and processed meat were reversed in the FQS scoring system to resolve this problem.

To the best of our knowledge, this report is the first study that has examined the association between FQS and cardiovascular risk factors. However, this study had some limitations. First, although we tried to take all possible confounders into account, controlling for all residual confounding factors in our study, as in all observational studies, was not possible. Another limitation was probable misclassification of individuals due to the use of semi-quantitative FFQ to collect dietary data. Finally, since our study was only performed in women aged 20–50 years, these findings are not generalizable.

In conclusion, these results showed that FQS was not significantly associated with CVD risk factors among women. Prospective studies are warranted to confirm whether there is a casual relationship between FQS and CVD risk factors.

## Competing interests

The authors report no conflicts of interest.

## Ethical approval

This study approved by Iran National Institute for Medical Research Development (NIMAD) (No.964124).

## Funding

This study is supported by Iran National Institute for Medical Research Development (NIMAD) (No.964124) and Tehran University of Medical Sciences.

## Acknowledgments

We would like to express our gratitude to Dr. Mohammad Shariati, Vice Chancellor for Health Affairs, Zahra Beygom Aghamiri, secretary of the Health Research Council of Tehran University of Medical Sciences, and all staff members of subsidiary health centers in Tehran, Iran..
